# Large overjet as a risk factor of traumatic dental injuries: a prospective longitudinal study

**DOI:** 10.1186/s40510-020-00341-5

**Published:** 2020-11-09

**Authors:** Jean-Paul Schatz, Enrico Ostini, Magnus Hakeberg, Stavros Kiliaridis

**Affiliations:** 1 Department of Orthodontics, Dental School of Medicine, University of Geneva, Geneva, Switzerland; 2 Department of Behavioral and Community Dentistry, Institute of Odontology, The Sahlgrenska Academy, University of Gothenburg, Gothenburg, Sweden

**Keywords:** Overjet, Dental injuries, Risk factor

## Abstract

**Purpose:**

The aim of this longitudinal prospective study was to evaluate if schoolchildren with large overjet experience a greater risk of traumatic dental injuries (TDI) compared to children with normal or small overjet.

**Methods:**

A sample of children aged 6‑13 years was prospectively evaluated after 1 year: from the initial sample, data concerning trauma cases of 1413 children were collected to determine the number and types of injuries, the influence of overjet on the risk of TDI, and the relationships between trauma, age, and gender.

**Results:**

The observed prevalence of trauma was higher for boys, with the largest frequency between the ages of 8 to 12 years: 67.9% of all injuries were hard tissue injuries and 32.1% subluxation and luxation injuries. Children with an overjet of 6 mm or more showed a statistically increased risk of getting trauma [RR = 3.37 with CI (1.81; 6.27)].

**Conclusion:**

In this prospective study, overjet stood out among variables as the most significant risk factor of TDI: an increased overjet of 6 mm or more had a major impact on the risk of trauma, which would speak in favor of early orthodontic correction of an increased overjet to reduce the prevalence of dental trauma

## Introduction

Orthodontic treatment indications are based on morphological descriptions of malocclusions which may influence oral health or relate treatment needs and motivation to esthetics, functional, or psychosocial needs [1]. Large overjet is among the occlusal deviations clearly identified as orthodontic treatment indications in the mixed dentition [1] and its early reduction has been recommended as an orthodontic measure to prevent tooth damage [2].

Epidemiologic investigations on traumatic dental injuries show prevalence ranges from 4 to 30% [3–5]. Most reports agree on specific sex and age distribution or seasonal variations, and they also suggest that increased overjet and inadequate lip coverage might be predisposing factors to traumatic injuries of the upper anterior teeth [6–10].

The relationship between overjet and traumatic injuries has been often investigated, leading to contradictory findings. While some studies found no correlation between overjet size and anterior tooth trauma [11, 12], others clearly reported an increasing risk of traumatic injuries with increasing overjet [7, 13, 14]: to date, there are only case-control or cross-sectional studies but no longitudinal panel surveys investigating the relation between excessive overjet and traumatic dental injuries.

The aim of this prospective study, collecting various epidemiological data on traumatic dental injuries in a Swiss population, was to evaluate if a specific sample population of schoolchildren with large overjet experience a greater risk of tooth damage after trauma compared to children with normal or small overjet, and therefore could benefit from trauma-preventive effect of early overjet correction.

## Subjects and methods

A sample of 1900 children aged 6 to 13 years was examined during routine consultations yearly performed by the Geneva Dental School Service. Eight schools were randomly stratified from a pool of Geneva public schools for the purpose of the research: the sample, including 1000 boys and 900 girls and accounting for 7.5% of all school children in Geneva’s primary public schools, was used in a previous cross-sectional study [10].

Information was collected through a cross-sectional examination in all age groups of children attending primary schools and they were seen by the same investigator. During the course of this routine control, special attention was devoted by the observer to discovering and recording any dental injuries sustained during the previous year, in order to define prevalence figures in each age group. Information was collected and processed anonymously, according to Swiss Federal Law on Research in Humans, and statement rules defined by the Geneva State Committee on Ethics in Research. The project was supported by a grant from the Swiss National Fund for Scientific Research (grant No.59485).

A special registration was introduced to record the following information:

- Patient history, name, age, sex, living conditions (urban or suburban areas), and grade level
- Type of dental injuries
- Overjet, measured parallel to the occlusal plane, to the nearest half millimeter, as the distance from the incisal edge of the most labial maxillary central incisor to the most labial mandibular central incisor

The measurements were collected by the same certified specialist in Orthodontics, with 10 year’s clinical experience and a completed training in malocclusion assessment based on intraoral measurements of morphological malocclusion traits. Measurement of OJ was made twice, once at the beginning and one at the end of clinical examination: the average absolute difference was calculated at 0.13 (SD = 0.09), the average standard difference at 0.06 (SD = 0.12), the individual standard deviation being 0.03.

All parents were informed about the dental status of their children at the time of examination—for the necessity of restorative treatment as for the presence of any orthodontic treatment indications—through a specific report file.

From this initial sample, 372 children who belonged to the older age group were not included in the prospective evaluation as, at the end of elementary primary school period, they were supposed to move to the first grade of secondary schools. Thus, the remaining 1528 children were intended to be enrolled in the prospective evaluation 1 year later. Finally, 1413 children could participate at the second examination, since some children were absent the day of the reevaluation, usually for medical or personal reasons, or they have moved to another school, i.e., with a dropout rate of 7.4%.

Re-examinations were made 1 year after the initial consultation by the same clinician to disclose new injury cases, and children in active orthodontic treatment were not included in the re-evaluation sample.

Data concerning the trauma cases were collected through a semi-structured interview: injuries were divided into hard tissue or luxation injuries. Hard tissue injuries were defined as enamel fracture or enamel-dentine fracture, with or without pulp exposure (as demonstrated by history of pulp extirpation). Luxation injuries were defined by the following criteria:

- Subluxation, loosening with no displacement of the tooth
- Intrusion, forced impaction of the tooth into the alveolar socket
- Extrusion, partial displacement of the tooth out of the socket
- Lateral luxation, forced movement of the tooth in a lateral direction
- Exarticulation, total luxation of the tooth

Each maxillary and mandibular tooth was scored for presence and type of traumatic injury according to the NIDR index, as stated in the Third National Health and Nutrition Examination survey in the USA [15]: the data are based on clinical non-radiographic evidence of the tooth injury and the treatment received, including a positive history obtained from each subject. The frequency of subjects with increased OJ, with a cut-off point of 6 mm, according to Järvinen [16], was calculated for the sub-samples of children with and without incisor trauma.

### Statistical analysis

The statistical analysis was performed using the chi-square test, to measure how expected counts compare to observed data, the *t* test, to evaluate the statistical significant difference between the two groups, one-way analysis of variance, and logistic regression for assessing risk indicators, such as gender, age, and overjet in relation to dental trauma. The significance level was set at *P* < 0.05; the SPSS version 14 was used as the statistical computer software (SPSS, Inc., Chicago, IL, USA).

## Results

### Initial

The population incorporated in this prospective study comprised 1413 children, 723 boys and 690 girls, seen in recall 1 year after initial examination, and representing 5.6% of all scholarized children in Geneva’s primary public schools.

Their age varied between 7 and 14 years for both boys and girls.

At the time of initial consultation, dental traumatic injuries have been recorded in 172 children, showing that 16.1% of boys and 12.1% of the girls of the sample experienced previous dental trauma. Among them, 53 cases were observed in patients with an increased OJ (≥ 6 mm), leading to a 4.03 times higher risk of sustaining trauma compared to patients with normal overjet.

### Follow-up

After 1 year, traumatic injuries, concerning 104 injured teeth, were recorded in 76 children (43 boys; 33 girls). The boy/girl ratio is 1.34 is to 1.

The largest number of injuries was comprised between the ages of 8 and 12 years, almost equally distributed and quoting for 93.4% of all the injuries (Table 1). Statistical significance for the association between incidence of dental trauma and gender was tested using chi-square tests: this model, however, was not significant (*p* = 0.05), i.e., no age group had a higher risk of getting trauma as compared to the other age groups.

**Table 1:** Number of injuries, according to age groups

	Age								Total
	7.00	8.00	9.00	10.0	11.0	12.0	13.0	14.0	
TRAUMA									
No trauma during follow-up period									
Count	32	160	212	243	249	223	182	36	1337
Expected count	31	164	214	241	256	221	175	35	1337
Trauma during follow-up period									
Count	1	13	14	12	22	10	3	1	76
Expected count	1.8	9.3	12.2	13.7	14.6	12.5	10.0	2.0	76.0
Total									
Count	33	173	226	255	271	233	185	37	1413
Expected count	33	173	226	255	271	233	185	37	1413

The figures for age repartition were similar for boys and girls.

The 76 subjects with traumatized teeth have been grouped, based on the location of the trauma. The grouping showed that 88.5% of trauma involved the upper front teeth, while 11.5% involved lower front teeth: single traumatized incisors were found in 76% of the sample, while only three patients had 3 or more injured teeth. Very few patients (1.9%) had injuries affecting incisors of the maxillary and mandibular arches at the same time.

As many as 28.9% of the recorded injuries were enamel fractures, 39% were combined enamel/dentine fractures, while subluxations and luxation injuries accounted for 32.1% of the traumas. There were 10.5% of the sample showing injuries combining hard tissues and subluxation injuries at the same time. We could not observe any relationship between the age of the patient at the time of injury and the type of the trauma experienced by the children.

In the total sample seen for re-examination after 1 year, 98 patients—none of them being in active orthodontic treatment—had an overjet of 6 mm or more: among them, 14 children experienced a traumatic dental injury, showing that children with an overjet of 6 mm, or larger than 6 mm, were more prone to traumatic injuries (*p* < 0.001). There was a statistically significant increased risk of getting trauma if having an overjet of 6 mm or more, the relative risk being RR = 3.37 with CI (1.81; 6.27) (Table 2).

**Table 2:** Risk estimate

	Value	95% Confidence interval	
		Lower	Upper
Odds ratio for TRAUMA02 (no trauma during follow-up period/trauma during follow-up period)	3368	1811	6265

## Discussion

The results of our prospective study confirm that children and adolescents with large OJ have an increased risk of traumatic dental injuries. In the total sample of 1413 patients seen for follow-up examination after 1 year, 76 patients experienced a traumatic injury. Among the 98 children from the total sample showing an overjet of 6 mm or more, 14 patients experienced a traumatic dental injury, showing a statistically significant increased risk after trauma, the relative risk being RR = 3.37. We found no gender difference among the groups but observed that almost a third of all traumas (29%) was seen in the 11 years old group age, while 76% of trauma were seen between the age of 9 and 12 years.

The relationship between overjet and traumatic dental injury has been often investigated, showing conflicting results among studies: nevertheless, Nguyen [9] concluded in his systematic review that children with an OJ larger than 3 mm are twice as much at risk of injury than those with an OJ of less than 3 mm. However, a cut-off point of 3 mm is somewhat questionable: when looking at the percentage of the US population with occlusal contact discrepancies, an OJ of 3‑4 mm concerns more than 40% of children under 17 years of age [17]. Bearing in mind the prevalence figures of traumatic dental injuries—which may affect almost one child out of two reaching 15 years of age—the inclusion of so many children in a study sample could dilute the results and underestimate the impact of very large OJ on the rate of traumas. When addressing this topic, Arraj et al. [18] showed that, within the same studies, children aged 12 years “have an OR of 5.19 when the overjet threshold was larger than 3 mm, but the OR was 1.81 when the OJ threshold of > 5 mm was applied.”

As yet, the possible trauma-preventive effect of early overjet correction remains largely controversial [19, 20]: some authors questioned the effectiveness of overjet reduction or its timing as it relates to injury [19–21], while others found that an early treatment of Cl.II, when associated with an overjet reduction, might have some favorable effect on the incidence of trauma [8]. The debate on the rationale of procedures aiming at the reduction of OJ to decrease the risk of traumatic dental injury was addressed by Koroluk et al. [20] in their study on incisor trauma incidence in a group of patients included in a clinical trial of 2-phase orthodontic treatment of Cl II malocclusions. The authors found no significant difference between the group treated early—with a headgear or a functional appliance—and the group in which treatment was initiated in permanent dentition. While considering their sample as too small to draw firm conclusions, they concluded that to prevent incisor trauma, any early treatment should be undertaken just after eruption of anterior teeth. Moreover, they stated that the increased cost of early overjet reduction, as a second phase in the permanent dentition is needed to achieve proper occlusal outcome, does not support a 2-phase orthodontic approach. On the opposite, Artun et al. [8] found that, as the peak of incisor trauma may not be reached before the age of 9 years, early correction of severely proclined incisors could prove beneficial with very limited additional costs.

The conclusions of Koroluk et al. were later endorsed by two different RC trials on early versus late Cl II treatment [21, 22], each of them demonstrating no correlations between OJ reduction and decrease of the risk of tooth trauma. However, and as stated in the UNC investigation [21], sample size calculations were not specifically meant to evaluate the correlation between OJ reduction and traumatic dental injuries. Due to low prevalence figures of tooth traumas, the samples incorporated in the RC trials should have incorporated much more patients to show significance. A recent Cochrane systematic review [2] compelled the evidence included in these three RC trials, showing a reduction of traumatic dental injuries in the patients of the early treatment group treated either by functional appliances or headgear therapy, without any significant statistical difference between the two different treatment approaches. However, their authors addressed some issues of these RC trials: high risk of bias, lack of sample size calculation, and extremely large confidence intervals leading them to consider that the findings, although somewhat encouraging, “should be interpreted in relation to the high level of uncertainty.”

Arraj et al. [18] also tried to compel evidence between increased overjet and tooth traumas; however, no clinical trials or prospective studies were identified for inclusion, and their review pooled studies with different thresholds of OJ and described no methodological quality assessment reports. In our prospective longitudinal study, on the contrary, the measured relative risk gives an information about cause-effect relationships, and exclude the possible recall bias by collecting data prospectively and following change over time in particular individuals within the cohort. Therefore, our results clearly demonstrate that an increased OJ lead to an elevated risk of traumatic dental injuries: bearing in mind the efficacy of the appliances used to reduce OJ by use of activator-type or headgear appliances demonstrated in these RC trials, we can conclude that interceptive orthodontic treatments to reduce OJ size would definitely lower the risk of tooth traumas. A possible limitation of our study is related to the use of a semi-structured interview to collect information on traumatic injuries, without radiographic examination. Therefore, some injuries such as root fractures may not have been recorded, or mislabeled as some luxation injuries, particularly extrusion and lateral luxation, could be confused using the structured interview mode. However, the low prevalence figures of traumatic injuries in a population of young children aged 7 to 13 years implies that such shortcomings should not affect the general conclusions of our study.

The concept of lowering the risk of trauma by reducing the OJ was also challenged by two other assumptions, one being that OJ reduction should be achieved much earlier to be effective—shortly after the eruption of incisors–the other that the cost induced by a first early phase of treatment would not be justified to avoid traumatic dental injuries, most of patients experiencing only enamel or dentine fractures showing good prognosis and requiring minimal treatment.

The prevalence figures of tooth traumas show they may affect 20% of the children population in the mixed dentition phase [3, 7]: in most epidemiologic reports, they seem to peak between the age of 10 to 12 years of age and considering a reduction of OJ as inefficient due to the timing of an early treatment is an invalid assumption. When looking at the results of the RC trials already mentioned, children of the early Cl.II treatment group, where growth modification was expected, all showed subsequent OJ reduction, stressing a clear indication for preventive measures at the period of peak incidence of traumatic dental injuries.

Moreover, in a 2-year prospective study, Glendor et al. [23] have shown that direct and indirect costs of tooth traumas may be much larger than previously agreed upon, especially when dealing with luxation injuries or complicated dental traumas, where resorptive processes due to periodontal ligament injuries can ultimately lead to the loss of anterior teeth. These data on the long-term prospective costs of traumatic dental injuries do not support the statement of Koroluk et al. [20] that “the policy to reduce the incidence of trauma in all children with excessive overjets would be financially prohibitive.”

## Conclusions

The results of this prospective study confirmed most of the results from earlier research dealing with epidemiological factors of dental injuries to the permanent dentition. Specifically designed to evaluate the risk of traumatic dental injuries in children with increased overjet, they showed that, of all the variables analyzed, overjet stood out as the most significant factor with a statistically significant increased risk of tooth trauma after traumatic injuries. An increased overjet of 6 mm or more has a major impact on the risk of trauma, which would speak in favor of early orthodontic correction of an increased overjet to reduce the prevalence of traumatic dental injuries among our children.

## Data Availability

The datasets generated and/or analyzed during the current study are not publicly available due to the Swiss Federal Law on Data Protection, but are available from the corresponding author on reasonable request.
